# Assessing Loneliness among Adults Receiving Outpatient Treatment with Medication for Opioid Use Disorder (MOUD)

**DOI:** 10.3390/ijerph192013481

**Published:** 2022-10-18

**Authors:** Rosalina Mills, Keith J. Zullig, Laurie A. Theeke, Laura R. Lander, Gerry R. Hobbs, Johnathan Herczyk, Stephen M. Davis

**Affiliations:** 1 Department of Social and Behavioral Sciences, School of Public Health, West Virginia University, Morgantown, WV 26506, USA; 2 School of Nursing, George Washington University, Washington, DC 20052, USA; 3 Department of Behavioral Medicine and Psychiatry, Rockefeller Neurosciences Institute, School of Medicine, West Virginia University, Morgantown, WV 26506, USA; 4 Department of Statistics, West Virginia University, Morgantown, WV 26506, USA; 5 Department of Health Policy, Management, & Leadership, School of Public Health, West Virginia University, Morgantown, WV 26506, USA

**Keywords:** medication for opioid use disorder outpatient therapy, treatment, intervention, loneliness, R-UCLA

## Abstract

Loneliness is a significant risk factor for substance use, however, impacts of treatments on loneliness are relatively unexplored. Living in a rural location is a greater risk factor for loneliness. This study examined data from a quasi-experimental study in rural Appalachia, comparing the effectiveness of Mindfulness-Based Relapse Prevention (MBRP) versus Treatment as Usual (TAU) among adults receiving MOUD in outpatient therapy. Our objective was to determine whether observed reductions in self-reported craving, anxiety, depression, and increased perceived mindfulness would also improve loneliness reports. Eighty participants (n = 35 MBRP; n = 45 TAU) were included in the analysis from a group-based Comprehensive Opioid Addiction Treatment program. Outcomes tracked included craving, anxiety, depression, mindfulness, and loneliness as measured by the Revised UCLA Loneliness Scale (R-UCLA). A linear mixed model ANOVA determined the significance of the treatments on changes in loneliness scores at baseline, 12 weeks, 24 weeks, and 36 weeks post-recruitment. Both groups reported significantly reduced loneliness over the course of the study (F = 16.07, *p* < 0.01), however there were no significant differences between groups. Loneliness was also significantly positively (*p* < 0.01) correlated with anxiety (0.66), depression (0.59), and craving (0.38), and significantly (*p* < 0.01) inversely correlated (−0.52) with mindfulness. Results suggest that participation in MOUD group-based outpatient therapy has the potential to diminish loneliness and associated poor psychological outcomes. Thus, it is possible that a more targeted intervention for loneliness would further diminish loneliness, which is important as loneliness is linked to risk for relapse.

## 1. Introduction

Substance use disorder (SUD) is prevalent in the United States and is defined as problematic and disordered use of substances such as alcohol, cannabis, cocaine, heroin, hallucinogens, inhalants, prescription opioids, sedatives, stimulants, and/or other drugs, according to the Diagnostic and Statistical Manual of Mental Disorders, Fifth Edition (DSM-5) criteria [1,2]. It is currently estimated that 20 million people aged 12 and older struggle with substance use disorders in the United States [3]. Furthermore, the use of opioids has become a national health crisis with over 80,000 Americans dying from opioid-related overdoses in 2021 [4]. Hence, there is a continuing need for research and treatments targeting potential risk factors for opioid use disorder (OUD), which is defined by DSM-5 diagnostic criteria as the disordered use of a prescription opioid, heroin, or both [5].

Existing literature has shown that Americans with psychological problems or psychiatric diagnoses are more likely to develop SUD and use opioids than Americans without these illnesses [6,7,8]. Loneliness is a significant stressor that has been consistently linked to negative physical, social, and psychological health outcomes [9,10,11]; including depression and anxiety, as well as with the use of alcohol, cigarettes and other substances [12,13,14,15]. Although loneliness prevalence varies by country and age group, it is typically highest among adults [16]. The national prevalence of loneliness in the United States is estimated to be between 11–22% for adults [17,18], and 35% for adults aged 45 and older [19]. One U.S. study found that 76% of sampled community-dwelling adults reported moderate or high levels of loneliness [20].

It is important to distinguish loneliness from social isolation. Social isolation is the lack of social contact and regular human interaction, while loneliness is the distressing feeling associated with being alone or isolated [21]. It is possible for people to be socially isolated and not lonely; contrarily, it is also possible to feel lonely while in the company of others. Loneliness and social isolation have both been associated with negative outcomes, such as mortality and mood and anxiety disorders [19,22,23]. While literature studying the relationship between social isolation and loneliness is sparse, an increase in social isolation, or a reduction in social networks, is a predictor of loneliness [24].

Loneliness has already been identified as a risk factor for opioid use [25,26,27]; and associated with relapse in people with OUD, women with depression and in treatment for OUD, and for people who use methamphetamine [26,28,29]. Thus, it may be critical to assess for and address loneliness as part of SUD treatment, yet this is not part of routine care for people with SUD [26,30]. Although mental health treatments that target loneliness have been developed [31,32], a recent systematic review identified only nine longitudinal studies that investigated loneliness in SUD treatment [13], and even fewer highlighted a need to investigate interventions for loneliness during OUD treatment [13]. Given the limited studies on loneliness, mindfulness and SUD treatment, there is a continuing need to study loneliness and its impact on recovery among those receiving treatment for OUD. In addition, these prior studies were predominantly conducted in urban areas, leaving a significant gap in the literature when seeking to understand the impact of loneliness on people who live in rural areas like Appalachia, a known area of extreme disparity in SUD.

### Rurality, Loneliness, and SUD

Appalachia is a rural region of the United States spanning 205,000 square miles, from New York State to Mississippi [33]. Appalachia has also been disproportionately affected by opioid overdoses and overdose death rates compared to the rest of the United States [33]. West Virginia, in particular, has the highest drug overdose deaths per capita, with rates peaking at 52.8 per 100,000 people in 2019 [34]. The highest proportion of these overdose deaths was from opioid misuse [34]. COVID-19 has reversed any effects of overdose reduction; recent estimates show overdose death increased from 2019 to 2020 by 45% in West Virginia [35].

West Virginia’s rural characteristics may also play a role in barriers to adequate healthcare. Nearly half of West Virginia’s counties do not provide waivers to prescribe buprenorphine, an evidence-based treatment for OUD [36]. Living in a rural location is also a substantially greater risk factor for social isolation and loneliness [37,38]. Despite these facts, there is a dearth of research on loneliness in substance-using, rural populations, and especially Appalachian populations.

The current study is a secondary analysis of data examining loneliness levels among adults receiving Medication for Opioid Use Disorder (MOUD) in outpatient therapy in a rural Appalachian state as part of an intervention testing the effectiveness of Mindfulness-Based Relapse Prevention (MBRP). Conducted over a period of 36 weeks, the intervention from which these data are derived demonstrated significantly reduced self-reported craving, anxiety, and depression as perceived mindfulness increased among MBRP/MOUD participants when compared to treatment as usual (TAU) cognitive behavioral therapy MOUD participants [39]. The intervention was not designed with the intent to address loneliness among participants, nor has there been any empirical investigation into an intervention’s potential impact on loneliness. However, there is a clear theoretical premise for such an investigation given previous research connecting loneliness to SUD.

Examining depression and anxiety is also important for the purposes of this study, given that these factors have been found to be prevalent in rural populations [40], and are also associated with loneliness and OUD [22,41]. Mood disorders often co-occur with SUD [41], therefore the results from the current study are more robust by also measuring depression and anxiety in addition to loneliness.

Existing evidence suggests that craving may also potentially influence substance use, specifically in OUD treatment populations [42]. Craving symptoms have been found to be associated with depression, anxiety, and negative social exchanges [42]. The importance of social support in the context of OUD treatment is highlighted by evidence demonstrating that daily positive social exchanges, that is, social support and positive interactions with others, helped reduce cravings experienced by patients in OUD treatment [42], providing additional evidence to study loneliness during OUD treatment. Thus, craving was included in the study as an indicator of future substance use, and as a factor that may be influenced by loneliness.

Mindfulness is also an important factor to consider when examining loneliness and OUD treatment. Mindfulness interventions specific to SUD address the relapse cycle by cultivating the awareness of triggers, attending mindfully to the discomforts the triggers elicited, and teaching targeted skills to cope with craving, thus facilitating the recovery process [43,44].

Systemic reviews, as well as individual randomized controlled trials, have shown that mindfulness-based interventions can significantly reduce loneliness [45,46,47,48]. Mindfulness training has been demonstrated to reduce loneliness in participants throughout the course of 6 to 8-week interventions [47]. However, when examined in the context of MOUD treatment, recent research suggests loneliness and other indicators of mental health (e.g., depression, anxiety, etc.) and well-being were not significantly reduced by MOUD alone after the first 6 months of treatment [49]. This research highlights a clear need for behavioral therapy and support in tandem with MOUD during treatment, such as mindfulness.

Thus, our study objective was to determine whether loneliness levels would improve among adults in MOUD outpatient treatment also receiving behavioral therapy. Our overall hypothesis was that over the course of the study, as participants engaged in the recovery process addressing psychological processes associated with OUD (e.g., anxiety, depression, and craving), reports of loneliness would also decrease. Anxiety, depression, and craving were included in the study owing to their relationship to loneliness in the literature. However, given the preliminary nature of the study, no hypotheses were made regarding between-group differences in loneliness reports.

## 2. Materials and Methods

Data were derived from a study conducted from September 2017–December 2019 that included participants recruited during the intermediate stage of treatment (patients with at least 90 consecutive days substance free) from a large, Mid-Atlantic university’s Comprehensive Opioid Addiction Treatment (COAT) program. Project investigators met with interested and eligible participants to describe the study, administer consent, and conduct baseline data assessments. Participants were then given the option to enroll in MBRP plus MOUD or remain in TAU MOUD. MBRP/MOUD group participants were assigned to attend bi-weekly 60-min group therapy sessions for 24 weeks. For a full description of the intervention, see Zullig et al. [39]. The referent university’s Institutional Review Board approved this study.

All analyses were conducted in SAS version 9.4. Descriptive statistics were first calculated followed by a linear mixed model ANOVA where fixed effects are reported in the results. For this analysis, we were specifically interested in determining whether loneliness levels would improve among intervention participants. Linear mixed models (also called multilevel models) are a method for analyzing data that are non-independent, multilevel/hierarchical, and longitudinal, which allowed us to explore the difference between effects within and between groups. Linear mixed models effectively use all of the available data to estimate change over time and is a preferred method to last value carried forward methods often used in intent-to-treat analyses. The analysis controlled for the demographic variables age, sex, marital status, education levels, employment status, and insurance specified a priori. We also used ANOVA to examine the correlations between the changes in loneliness at 36 weeks for the baseline demographic variables. Cohen’s d was used to calculate effect sizes to determine the magnitude of statistically significant findings. Lastly, we performed a series of correlations at baseline to assess the strength of the association between perceived loneliness and negative psychological health outcomes [9,10,11] including depression and anxiety. An alpha level of 0.05 was used to determine the statistical significance of all analyses.

### Measures

Study outcomes were participants’ self-reported craving, depression, and anxiety levels; mindfulness; and loneliness. All measures were administered at baseline, after 12 weeks, post-intervention (24 weeks), and again 36 weeks post-intervention to MBRP/MOUD and TAU/MOUD study participants.

Craving symptoms. Craving symptoms were measured by the 14-item Desire for Drugs Questionnaire (DDQ). Response options are (a) strongly disagree, (b) disagree, (c) undecided, (d) agree, and (e) strongly agree with values from 1 (strongly disagree) to 5 (strongly agree) assigned. The sum of the response values (range 14–70) was the outcome of interest with higher values indicative of greater cravings. The DDQ has previously demonstrated acceptable validity and internal consistency reliability with estimates above 0.80 [50]. The baseline internal consistency estimate for the DDQ in this study was 0.78.

Depression. Depression was assessed using the 5-item Overall Depression Severity and Impairment Scale (ODSIS). Each item has 4 response options that are summed (range 0–20) with a total score of 8 or higher used to determine a depression diagnosis (correctly classifies over 80%). The scale has demonstrated acceptable validity and internal consistency reliability with estimates exceeding 0.91 [51]. The baseline internal consistency estimate for the ODSIS in this study was 0.89.

Anxiety. Anxiety was assessed using the 5-item Overall Anxiety Severity and Impairment Scale (OASIS). Each OASIS item has 4 response options that are summed (range 0–20) with a total score of 8 or higher used to determine an anxiety diagnosis (correctly classifies over 80%). The scale has demonstrated acceptable validity and internal consistency reliability with estimates exceeding 0.80 [52,53]. The baseline internal consistency estimate for the OASIS in this study was 0.92.

Mindfulness. Mindfulness was measured with the 39-item self-report 5-Facet Mindfulness Questionnaire (FFMQ). Response options are (a) never or very rarely true, (b) rarely true, (c) sometimes true, (d) often true, and (e) very often or always true with values from 1 (never or very rarely true) to 5 (very often or always true). For this study, the sum of the responses for the total scale were divided by 5 (range 1–5) with higher values indicating greater mindfulness. The FFMQ has demonstrated adequate validity and internal consistency with estimates ranging from 0.75 to 0.91 for the five subscales in prior research [54]. The baseline internal consistency estimate for the FFMQ total scale in this study was 0.89.

Loneliness. Loneliness was assessed using the 20-item Revised-UCLA Loneliness Scale (R-UCLA) [55]. This scale is a self-report Likert scale, with 4 answer options for each item: (1) never, (2) sometimes, (3) often, and (4) always. Nine of these 20 items are reverse-scored; the minimum score for this scale is 20, while the maximum is 80, with lower values indicating improved perceptions of loneliness. The R-UCLA scale is considered the gold standard for measuring loneliness given (1) its ease of administration, (2) acceptable reliability and validity [56], and (3) ability to measure change over time [56,57]. The prevalence of loneliness in many studies using the R-UCLA indicate feeling lonely “at least some of the time” [58], with a threshold of 44 or greater for the full 20-item UCLA scale [59]. Therefore, it is the scale most often used in studies of loneliness, particularly as those pertaining to SUD [13]. The baseline internal consistency estimate for the R-UCLA in this study was 0.92.

## 3. Results

**Figure 1:**
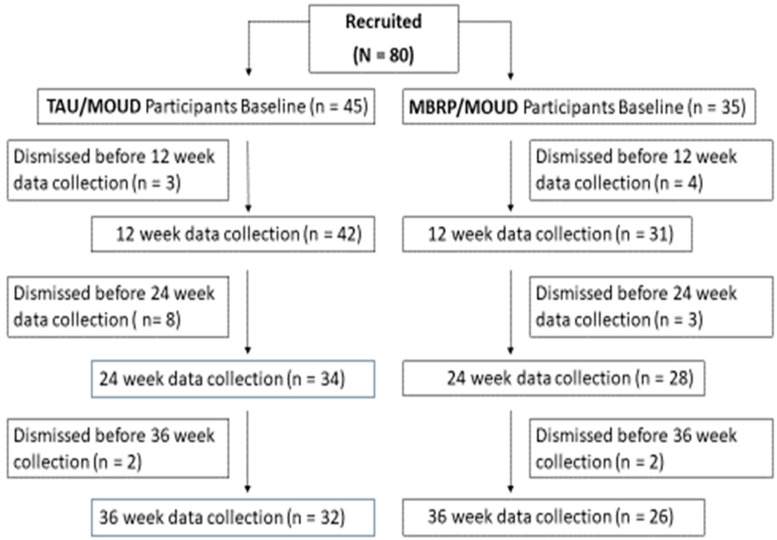
Intervention flowchart.

A total of 80 participants were included in the analysis (MBRP/MOUD, n = 35; TAU/MOUD, n = 45). The intervention flow chart is provided in Figure 1 and group baseline demographics are located in Table 1. The overall sample mean age was 36.3 (SD = 8.7).

For categorical characteristics in Table 1, no significant differences were detected at baseline between MBRP/MOUD and TAU/MOUD participants within marital status (*p* = 0.63), sex (*p* = 0.12), employment (*p* = 0.95), education (*p* = 0.10), or insurance (*p* = 0.59). A t-test comparing groups in age revealed MBRP/MOUD group participants were statistically significantly (*p* = 0.02) younger (M = 34.9, SD = 6.9) than TAU group participants (M = 37.3, SD = 10.3). However, the effect size for this difference was small (Cohen’s d = 0.21), suggesting this difference was not practically important.

**Figure 2:**
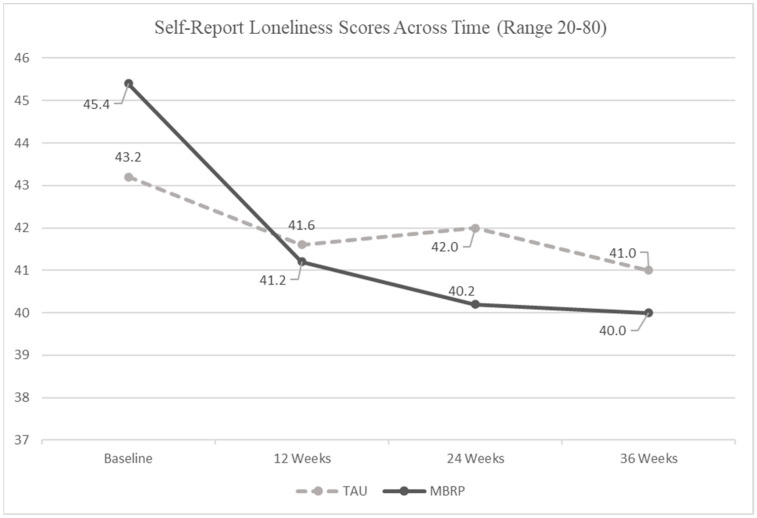
Self-Report Loneliness Scores by Group across the 36 Week Intervention from the Linear Mixed Model.

Results of the regression analysis are located in Figure 2. Results suggest both MBRP/MOUD and TAU/MOUD groups reported significantly reduced loneliness over the course of the study (F = 16.07, *p* < 0.0001, Cohen’s d = 0.20) even after controlling for the covariates. However, no significant differences in loneliness reports were detected in the interaction between weeks and groups over the 36-week time period (F = 0.88, *p* = 0.35).

Nevertheless, given the sharper reductions in reported loneliness scores observed among MBRP/MOUD participants in comparison to the TAU/MOUD participants between the baseline and 12-week data, we investigated the decline in loneliness across time in the MBRP/MOUD group and found significance (F = 13.83, *p* < 0.001). This exploratory result suggests loneliness decreased in the MBRP/MOUD group more than the TAU/MOUD group from baseline to 12 weeks.

ANOVA results examining the correlations between the changes in loneliness at 36 weeks for the baseline demographic variables yielded no statistically significant findings. The *p*-values for these analyses were 0.85 for age, 0.79 for sex, 0.09 employment, 0.48 for education, and 0.34 for insurance.

Loneliness was significantly positively (*p* < 0.01) correlated with anxiety, depression, and craving. Specifically, baseline correlation coefficients between loneliness and anxiety, depression, and craving were 0.66, 0.59, and 0.38, respectively. Loneliness and mindfulness were also significantly (*p* < 0.01) inversely correlated at baseline r = −0.52. However, when separated by intervention condition (i.e., MBRP/MOUD and TAU/MOUD), the associations between mindfulness and loneliness were stronger for individuals in the MBRP/MOUD group. For instance, the baseline correlation coefficient between loneliness and mindfulness in the MBRP/MOUD group was −0.59 (*p* < 0.01) in comparison to the TAU/MOUD group r = −0.39 (*p* < 0.05).

## 4. Discussion

Although loneliness has been identified as a possible risk factor for SUD or OUD [26,27] in cross-sectional studies and as a possible reason for relapse [25] in qualitative research, limited research has explored the longitudinal association between loneliness and its correlates among those receiving MOUD in outpatient treatment [13]. Moreover, we were able to locate only two longitudinal studies on outpatient OUD treatment. The first was conducted with urban adults receiving buprenorphine treatment and suggests that very lonely adults with substance use disorder may have more difficulty with cessation [60]. For instance, participants in their study with the highest levels of loneliness were most likely to have non-prescribed opioids present in their oral fluid or urine during drug testing [60]. The second was also conducted with participants in a large metropolitan city receiving MOUDs and no behavioral therapy and found loneliness was not significantly reduced after six months treatment [49].

Findings from the current study suggest both TAU/MOUD and MBRP/MOUD groups reported statistically significantly reduced perceptions of loneliness over the course of the intervention, however the effect was “small”. It is worth underlining that although the effect size was small, these results were found despite the fact that neither TAU/MOUD nor MBRP/MOUD were specifically designed with the intent of addressing loneliness among participants.

In addition, no significant differences in loneliness reports were detected in the interaction between weeks and groups. Nevertheless, additional exploratory analysis detected a significant intervention effect where loneliness decreased in the MBRP/MOUD group more sharply when compared to the TAU/MOUD group over the first 12 weeks. However, analyses examining the correlations between the changes in loneliness at 36 weeks for the baseline demographic variables yielded no statistically significant findings. The results suggest age, sex, employment status, education levels, and insurance status were not important confounders.

This is not the first study to postulate that a mindfulness intervention has potential to help with loneliness. A recent systematic review with meta-analysis on mindfulness as a treatment for loneliness concluded that mindfulness intervention was useful in relieving loneliness among participants with no mental health conditions [47]. In addition, Creswell and colleagues reported that mindfulness based programs can help with loneliness and isolation in adults [45]. However, the current study may be the first to document that loneliness can be diminished in people with OUD with concurrent psychological problems where between 43% to 49% of the sample (depending on group) reported anxiety above the clinical threshold and between 38% to 65% of the sample reported depression above the clinical threshold [39].

The higher initial levels of reported loneliness in this population are congruent with prior scientific literature indicating that people with substance use disorder experience loneliness [28]. This knowledge paired with the findings of statistically significantly lower levels of loneliness at 36-weeks post recruitment is consistent with the research suggesting that engaging in social changes during the early phase of OUD treatment may lead to loneliness [13,60,61].

This study presents new information about loneliness and its relation to healing in people with OUD. The information begins to fill a critical need for knowledge and could be used to inform designs for more precise interventions among individuals who experience loneliness and have SUD. Current treatment programs often encourage patients to avoid previous friendships or associations in order to avoid substance use triggers or opportunities [60]. This is a needed lifestyle change for successful recovery but programs do not always offer ways to combat the loneliness and isolation that can ensue when a person enters treatment. Acknowledging loneliness as a potential real problem that occurs when people with SUD try to heal will mean incorporating strategies that target loneliness and isolation into treatment programs.

It is also key to avoid conflating social isolation with loneliness when treating OUD. The unique constructs of isolation and loneliness require different treatment, plans to rebuild a supportive network and frequent contacts, and plans to address the maladaptive thinking that often accompanies loneliness. Recent research has shown that mindfulness training may be effective at reducing loneliness and increasing social contact for adults [46]. Therefore, although speculative, the reduction in perceived loneliness within both groups over the 36-week intervention may, in part, be attributed to the fostering of new social groups and increased social support that were created throughout the course of treatment. Loneliness may have improved over time in both groups due to the sharing of common experiences which fosters social supports in treatment groups. Group therapy has been recognized as the treatment of choice for SUD for decades owing to addiction being associated with depression, anxiety, isolation, denial, shame, and the need for social skills building [62].

The correlational findings are not surprising given that loneliness has been positively associated with both depression and anxiety in previous research, and is a known precursor to a variety of mental illnesses [63,64]. The correlations from the current study support the current narrative identifying loneliness as a predictor for anxiety and depression. As perceptions of loneliness decreased, so did reports of depression and anxiety. The inverse association between loneliness and mindfulness is also consistent with the expectation that increased perceived mindfulness would be associated with decreased, or improved, perceptions of loneliness in the present study, regardless of treatment [46].

This study is one of the first to offer information about the relationship between loneliness and drug craving. Prior studies have reported loneliness and drug cravings as independently associated with depression and anxiety [65]. In fact, loneliness and drug craving have typically been coupled together and studied as a combined effect on substance use, depression, and anxiety [65]. While there is a direct relationship between loneliness and substance use itself [13,30,65], the relationship between loneliness and drug craving alone is understudied. Future studies should include measures of loneliness, social connectedness, and social isolation in order to better understand the influence of each on use and relapse, as well as to further understand how the psychological construct of loneliness influences cravings. This is important since cravings are both psychological and physiological.

### Limitations

The study employed a quasi-experimental study design, and therefore, selection bias and confounding cannot be ruled out given that participants were not randomized to groups. A statistically significant difference between groups at baseline was detected for age, however age was not a significant predictor in the analysis. No other significant demographic differences were detected between groups at baseline. We also cannot definitively conclude that the OUD treatments caused the positive reductions in loneliness reports given the study design. Future randomized controlled trials are necessary to fully understand the effect of interventions targeting loneliness in this population. Our study sample also identified primarily as white, which while representative of the Appalachian region, it does limit generalizability to other populations. It is also possible that our sample size may have left the study somewhat underpowered. Quantitatively, MBRP/MOUD participants experienced approximately 2.5 times more improvement in loneliness from baseline to 12 weeks than TAU/MOUD participants and consistently better loneliness levels throughout the intervention. Studies with larger samples may have different conclusions [39].

## 5. Conclusions

Study results provide additional support to the literature suggesting that loneliness may be an important construct to address for individuals in MOUD treatment. The prevalence of loneliness in this study and in existing research [13] coupled with the dearth of empirical studies conducted in the context of MOUD treatment make it critical to continue this work. Novel interventions are needed for people with OUD so that they are implementable on a large scale. While interventions for loneliness in populations who use substances are sparse, some intervention studies have demonstrated success in diminishing loneliness in other populations. For example, interactive workshops and the LISTEN intervention demonstrated effectiveness in an elderly Appalachian population [31]. Other potential strategies include mindfulness training, social support interventions and social cognitive training [32,46]. Results from the current study suggest that testing the potential transferability of these successful strategies to populations who use substances offers future researchers potential avenues to explore. In addition, full consideration will be given on how these findings could inform the design of telehealth-based interventions. Future qualitative studies could provide additional insights into understanding what mattered most to people with OUD who experience loneliness.

Recognizing loneliness as a unique health risk worthy of assessment and intervention in this population and others will be key to treating people who experience loneliness. The current study was conducted pre-pandemic but it is important to note that there may be even a greater need to include loneliness when studying addiction due to increases in both addiction and loneliness reported during the COVID-19 pandemic [12,57,66].

## Figures and Tables

**Table 1:** MBRP Intervention Demographic Data by Group and Total (N = 80).

Demographic	TAU (n = 45)	MBRP (n = 35)	Total	*p*-Value
Marital Status				0.63
Single	28 (60.9%)	18 (39.1%)	46	
Married	8 (50.0%)	8 (50.0%)	16	
Divorced or Separating	9 (50.0%)	9 (50.0%)	18	
Sex				0.12
Male	24 (64.9%)	13 (35.1%)	37	
Female	21 (51.2%)	20 (48.9%)	41	
Other	0 (0.0%)	2 (100.0%)	2	
Race				n/a
White	45 (56.3%)	35 (43.8%)	80	
Employment				0.95
Full Time	18 (58.1%)	13 (41.9%)	31	
Part Time	8 (53.3%)	7 (46.7%)	15	
Unemployed	19 (55.9%)	15 (44.1%)	34	
Education				0.10
Did Not Finish High School	3 (37.5%)	5 (62.5%)	8	
High School Graduate/GED	30 (66.7%)	15 (33/3%)	45	
Some College or Greater	11 (40.1%)	16 (59.3%)	27	
Insurance				0.59
Medicaid	34 (52.3%)	31 (47.8%)	65	
Medicare	3 (0.6%)	2 (0.4%)	5	
Private	7 (70.0%)	3 (30.0%)	10	

TAU = Treatment as Usual; MBRP = Mindfulness Based Relapse Prevention (Intervention).

## Data Availability

The datasets used and/or analyzed during the current study are available from the corresponding author on reasonable request.

## References

[1] Grant B.F., Saha T.D., Ruan W.J., Goldstein R.B., Chou S.P., Jung J., Hasin D.S. (2016). Epidemiology of DSM-5 drug use disorder: Results from the National Epidemiologic Survey on Alcohol and Related Conditions—III. JAMA Psychiatry.

[2] McCabe S.E., West B.T., Jutkiewicz E.M., Boyd C.J. (2017). Multiple DSM-5 substance use disorders: A national study of US adults. Hum. Psychopharmacol. Clin. Experimental.

[3] National Center for Drug Abuse Statistics (2022). Drug Abuse Statistics. https://drugabusestatistics.org/.

[4] National Center for Health Statistics (2022). U.S. Overdose Deaths in 2021 Increased Half as Much as in 2020—But Are Still Up 15%. https://www.cdc.gov/nchs/pressroom/nchs_press_releases/2022/202205.htm.

[5] Jones C.M., McCance-Katz E.F. (2019). Co-occurring substance use and mental disorders among adults with opioid use disorder. Drug Alcohol Depend..

[6] Davis M.A., Lin L.A., Liu H., Sites B.D. (2017). Prescription opioid use among adults with mental health disorders in the United States. J. Am. Board Fam. Med..

[7] Kelly T.M., Daley D.C. (2013). Integrated treatment of substance use and psychiatric disorders. Soc. Work Public Health.

[8] Ross S., Peselow E. (2012). Co-Occurring Psychotic and Addictive Disorders. Clin. Neuropharmacol..

[9] Kelly D., Steiner A., Mazzei M., Baker R. (2019). Filling a void? The role of social enterprise in addressing social isolation and loneliness in rural communities. J. Rural. Stud..

[10] Luo Y., Hawkley L.C., Waite L.J., Cacioppo J.T. (2012). Loneliness, health, and mortality in old age: A national longitudinal study. Soc. Sci. Med..

[11] Tremeau F., Antonius D., Malaspina D., Goff D.C., Javitt D.C. (2016). Loneliness in schizophrenia and its possible correlates. An exploratory study. Psychiatry Res..

[12] Horigian V.E., Schmidt R.D., Feaster D.J. (2020). Loneliness, Mental Health, and Substance Use among US Young Adults during COVID-19. J. Psychoact. Drugs.

[13] Ingram I., Kelly P.J., Deane F.P., Baker A.L., Goh M.C.W., Raftery D.K., Dingle G.A. (2020). Loneliness among people with substance use problems: A narrative systematic review. Drug Alcohol. Rev..

[14] Stickley A., Koyanagi A., Koposov R., Schwab-Stone M., Ruchkin V. (2014). Loneliness and health risk behaviours among Russian and U.S. adolescents: A cross-sectional study. BMC Public Health.

[15] Wootton R.E., Greenstone H.S.R., Abdellaoui A., Denys D., Verweij K.J.H., Munafò M.R., Treur J.L. (2021). Bidirectional effects between loneliness, smoking and alcohol use: Evidence from a Mendelian randomization study. Addiction.

[16] Surkalim D.L., Luo M., Eres R., Gebel K., van Buskirk J., Bauman A., Ding D. (2022). The prevalence of loneliness across 113 countries: Systematic review and meta-analysis. BMJ.

[17] DiJulio B.L., Hamel L., Munana C., Brodie M. (2018). Loneliness and Social Isolation in the United States, the United Kingdom, and Japan: An International Survey.

[18] Kaiser Family Foundation, The Economist (2018). Survey on Loneliness and Social Isolation in the United States, the United Kingdom, and Japan. http://files.kff.org/attachment/Topline-Kaiser-Family-Foundation-The-Economist-Survey-on-Loneliness-and-Social-Isolation-in-the-United-States-the-United-Kingdom-and-Japan.

[19] National Academies of Sciences, Engineering, and Medicine (2020). Social Isolation and Loneliness in Older Adults: Opportunities for the Health Care System.

[20] Lee E.E., Depp C., Palmer B.W., Glorioso D., Daly R., Liu J., Jeste D.V. (2019). High prevalence and adverse health effects of loneliness in community-dwelling adults across the lifespan: Role of wisdom as a protective factor. Int. Psychogeriatr..

[21] NIH National Institutes on Aging (2021). Loneliness and Social Isolation—Tips for Staying Connected. National Institutes of Health. https://www.nia.nih.gov/health/loneliness-and-social-isolation-tips-staying-connected#:~:text=Loneliness%20is%20the%20distressing%20feeling,while%20being%20with%20other%20people.

[22] Barg F.K., Huss-Ashmore R., Wittink M.N., Murray G.F., Bogner H.R., Gallo J.J. (2006). A mixed methods approach to understanding loneliness and depression in older adults. J. Gerontol. Ser. B.

[23] Theeke L.A., Mallow J. (2013). Loneliness and quality of life in chronically ill rural older adults. Am. J. Nurs..

[24] Petersen J., Kaye J., Jacobs P.G., Quinones A., Dodge H., Arnold A., Thielke S. (2016). Longitudinal relationship between loneliness and social isolation in older adults: Results from the cardiovascular health study. J. Aging Health.

[25] Laudet A.B., Magura S., Vogel H.S., Knight E.L. (2004). Perceived reasons for substance misuse among persons with a psychiatric disorder. Am. J. Orthopsychiatry.

[26] Polenick C.A., Cotton B.P., Bryson W.C., Birditt K.S. (2019). Loneliness and Illicit Opioid Use Among Methadone Maintenance Treatment Patients. Subst. Use Misuse.

[27] Vyas M.V., Watt J.A., Yu A.Y., Straus S.E., Kapral M.K. (2021). The association between loneliness and medication use in older adults. Age Ageing.

[28] Johnson J.E., Schonbrun Y.C., Nargiso J.E., Kuo C.C., Shefner R.T., Williams C.A., Zlotnick C. (2013). “I know if I drink I won’t feel anything”: Substance use relapse among depressed women leaving prison. Int. J. Prison. Health.

[29] Newton T.F., De La Garza R., Kalechstein A.D., Tziortzis D., Jacobsen C.A. (2009). Theories of addiction: Methamphetamine users’ explanations for continuing drug use and relapse. Am. J. Addict..

[30] Hosseinbor M., Yassini Ardekani S.M., Bakhshani S., Bakhshani S. (2014). Emotional and social loneliness in individuals with and without substance dependence disorder. Int. J. High Risk Behav. Addict..

[31] Theeke L.A., Mallow J.A., Moore J., McBurney A., Rellick S., VanGilder R. (2016). Effectiveness of LISTEN on loneliness, neuroimmunological stress response, psychosocial functioning, quality of life, and physical health measures of chronic illness. Int. J. Nurs. Sci..

[32] Veronese N., Galvano D., D’Antiga F. (2021). Interventions for reducing loneliness: An umbrella review of intervention studies. Health Soc. Care Community.

[33] Meit M., Heffernan M., Tanenbaum E. (2019). Investigating the impact of the diseases of despair in Appalachia. J. Appalach. Health.

[34] Centers for Disease Control and Prevention (CDC) (2021). Drug Overdose Mortality by State. https://www.cdc.gov/nchs/pressroom/sosmap/drug_poisoning_mortality/drug_poisoning.htm.

[35] Department of Health and Human Resources (DHHR) (2020). West Virginia Experiences Increase in Overdose Deaths; Health Officials Emphasize Resources. https://dhhr.wv.gov/News/2021/Pages/West-Virginia-Experiences-Increase-in-Overdose-Deaths;-Health-Officials-Emphasize-Resources.aspx.

[36] Andrilla C.H.A., Moore T.E., Patterson D.G., Larson E.H. (2019). Geographic distribution of providers with a DEA waiver to prescribe buprenorphine for the treatment of opioid use disorder: A 5-year update. J. Rural. Health.

[37] Henning-Smith C., Ecklund A., Lahr M., Evenson A., Moscovice I., Kozhimannil K. (2018). Key Informant Perspectives on Rural Social Isolation and Loneliness. University of Minnesota Rural Health Research Center. http://rhrc.umn.edu/wp-content/uploads/2019/01/1539002382UMNpolicybriefKeyInformantPerspectivesonRuralSocialIsolationandLoneliness.pdf.

[38] Savikko N., Routasalo P., Tilvis R.S., Strandberg T.E., Pitkälä K.H. (2005). Predictors and subjective causes of loneliness in an aged population. Arch. Gerontol. Geriatr..

[39] Zullig K.J., Lander L.R., Tuscano M., Garland M., Hobbs G.R., Faulkenberry L. (2021). Testing Mindfulness-Based Relapse Prevention with medications for opioid use disorder among adults in outpatient therapy: A quasi-experimental study. Mindfulness.

[40] Friedman B., Conwell Y., Delavan R.L. (2007). Correlates of late-life major depression: A comparison of urban and rural primary care patients. Am. J. Geriatr. Psychiatry.

[41] Nkyi A.K., Ninnoni J.P.K. (2020). Depression, Purpose in Life, Loneliness and Anxiety Among Patients with Substance Use Disorders in Ankaful Psychiatric Hospital in Ghana. J. Psychosoc. Rehabil. Ment. Health.

[42] Knapp K.S., Bunce S.C., Brick T.R., Deneke E., Cleveland H.H. (2020). Daily associations among craving, affect, and social interactions in the lives of patients during residential opioid use disorder treatment. Psychol. Addict. Behav..

[43] Bowen S., Witkiewitz K., Dillworth T.M., Chawla N., Simpson T.L., Ostafin B., Larimer M.E., Blume A.W., Parks G.A., Marlatt G.A. (2006). Mindfulness meditation and substance use in an incarcerated population. Psychol. Addict. Behav..

[44] Bowen S., Witkiewitz K., Clifasefi S.L., Grow G., Chawla N., Hsu S.H., Carroll H.A., Harrop E., Collins S.E., Lustyk M.K. (2014). Relative efficacy of mindfulness-based relapse prevention, standard relapse prevention, and treatment as usual for substance use disorders: A randomized clinical trial. JAMA Psychiatry.

[45] Creswell J.D., Irwin M.R., Burklund L.J., Lieberman M.D., Arevalo J.M., Ma J., Cole S.W. (2012). Mindfulness-based stress reduction training reduces loneliness and pro-inflammatory gene expression in older adults: A small randomized controlled trial. Brain Behav. Immun..

[46] Lindsay E.K., Young S., Brown K.W., Smyth J.M., Creswell J.D. (2019). Mindfulness training reduces loneliness and increases social contact in a randomized controlled trial. Proc. Natl. Acad. Sci. USA.

[47] Teoh S.L., Letchumanan V., Lee L.H. (2021). Can mindfulness help to alleviate loneliness? A systematic review and meta-analysis. Front. Psychol..

[48] Zhang N., Fan F.M., Huang S.Y., Rodriguez M.A. (2018). Mindfulness training for loneliness among Chinese college students: A pilot randomized controlled trial. Int. J. Psychol..

[49] Hooker S.A., Lonergan-Cullum M., Levy R., Nissly T., Sherman M.D. (2021). Longitudinal assessment of mental health and well-being in patients being treated with medications for opioid use disorder in primary care. Addict. Behav. Rep..

[50] Franken I.H.A., Hendriks V.M., van den Brink W. (2002). Initial validation of two opiate craving questionnaires: The Obsessive Compulsive Drug Use Scale and the Desires for Drug Questionnaire. Addict. Behav..

[51] Bentley K.H., Gallagher M.W., Carl J.R., Barlow D.H. (2014). Development and validation of the Overall Depression Severity and Impairment Scale. Psychol. Assess..

[52] Campbell-Sills L., Norman S.B., Craske M.G., Sullivan G., Lang A.J., Chavira D.A., Bystritsky A., Sherbourne C., Roy-Byrne P., Stein M.B. (2009). Validation of a brief measure of anxiety-related severity and impairment: The Overall Anxiety Severity and Impairment Scale (OASIS). J. Affect. Disord..

[53] Norman S.B., Hami Cissell S., Means-Christensen A.J., Stein M.B. (2006). Development and validation of an overall anxiety severity and impairment scale (OASIS). Depress. Anxiety.

[54] Baer R.A., Smith G.T., Hopkins J., Krietemeyer J., Toney L. (2006). Using self-report assessment methods to explore facets of mindfulness. Assessment.

[55] Russell D. (1996). UCLA Loneliness Scale (Version 3): Reliability, validity, and factor structure. J. Personal. Assess..

[56] Russell D.W., Peplau L.A., Cutrona C.E. (1980). The revised UCLA Loneliness Scale: Concurrent and discriminant validity evidence. J. Personal. Soc. Psychol..

[57] Groarke J.M., Berry E., Graham-Wisener L., McKenna-Plumley P.E., McGlinchey E., Armour C. (2020). Loneliness in the UK during the COVID-19 pandemic: Cross-sectional results from the COVID-19 Psychological Wellbeing Study. PLoS ONE.

[58] Kuwert P., Knaevelsrud C., Pietrzak R.H. (2014). Loneliness among older veterans in the United States: Results from the National Health and Resilience in Veterans Study. Am. J. Geriatr. Psychiatry.

[59] Wilson C., Moulton B. (2010). Loneliness among Older Adults: A National Survey of Adults 45+. Knowledge Networks and Insight Policy Research.

[60] McDonagh J., Williams C.B., Oldfield B.J., Cruz-Jose D., Olson D.P. (2020). The Association of Loneliness and Non-prescribed Opioid Use in Patients With Opioid Use Disorder. J. Addict. Med..

[61] Ingram I., Kelly P.J., Deane F.P., Baker A.L., Dingle G.A. (2020). Perceptions of loneliness among people accessing treatment for substance use disorders. Drug Alcohol Rev..

[62] Center for Substance Abuse Treatment (CSAT) (2005). Substance Abuse Treatment: Group Therapy.

[63] Dziedzic B., Sarwa P., Kobos E., Sienkiewicz Z., Idzik A., Wysokiński M., Fidecki W. (2021). Loneliness and Depression among Polish High-School Students. Int. J. Environ. Res. Public Health.

[64] Erzen E., Çikrikci Ö. (2018). The effect of loneliness on depression: A meta-analysis. Int. J. Soc. Psychiatry.

[65] Chen X., Qiu N., Zhai L., Ren G. (2021). Anxiety, Loneliness, Drug Craving, and Depression Among Substance Abusers in Sichuan Province, China. Front. Pharmacol..

[66] Volkow N.D. (2020). Collision of the COVID-19 and addiction epidemics. Ann. Intern. Med..

